# COVID-19 Pandemic and Death Anxiety in Security Forces in Spain

**DOI:** 10.3390/ijerph17217760

**Published:** 2020-10-23

**Authors:** Cristina Lázaro-Pérez, José Ángel Martínez-López, José Gómez-Galán, María del Mar Fernández-Martínez

**Affiliations:** 1 Department of Sociology, University of Murcia, Campus Universitario, 11, 30100 Murcia, Spain; cristina.lazaro2@um.es; 2 Department of Social Work and Social Services, University of Murcia, Avda. Teniente Flomesta, 5, 30003 Murcia, Spain; jaml@um.es; 3 Department of Education, University of Extremadura, Avda. de Elvas, s/n, 06006 Badajoz, Spain; 4 College of Education, Ana G. Méndez University, Cupey Campus, San Juan, PR 00926, USA; 5 College of Education Sciences & College of Sociology, Social Work and Public Health, University of Huelva, Campus El Carmen, Avda. de las Fuerzas Armadas, s/n, 21007 Huelva, Spain; mar.fernandez@dstso.uhu.es

**Keywords:** death anxiety, burnout, police, armed forces, state security forces, occupational health

## Abstract

The pandemic caused by the SARS-CoV-2 coronavirus, which produces COVID-19 disease, has revealed to political and social circles a series of needs that have not yet been met. The workers of the State Security Forces and the Armed Forces have done an extraordinary job to try to alleviate the effects that the pandemic has had on the population and to return stability to the citizenry as much as possible. In this context, the following investigation is developed based on two objectives: (PO1) to know the level of anxiety in the face of death in these professionals; (PO2) to determine the predictive variables in the above-mentioned phenomenon. Professionals from all over Spain have participated in the study (n = 2079). From a quantitative perspective, a questionnaire was developed from the Collet–Lester death anxiety scale. The results show a total level of 69.2% in the scale, as well as some higher levels about the fear of death of others (82.1%) and the fear of the process of dying of others (78.2%). On the other hand, from the binary logistic regressions, four variables are evidenced that condition the risk of suffering death anxiety: (a) certainty of needing psychological treatment in the future; (b) absence of Individual Protection Equipment (PPE); (c) high levels of Emotional Exhaustion; (d) high levels of depersonalization—these last two come from the Maslach and Jackson Burnout scale. These data show a need for training and intervention in the emotional and psychological demands of the professionals of the Armed Forces and State Security Forces, as well as the obligation to develop a continuous dialogue with the institutions they represent to foster the feeling of belonging to them. It is essential, regardless of the serious consequences that the virus has caused, to understand the psychosocial and emotional demands of enforcement agents and to improve their occupational health.

## 1. Introduction

The three institutions that exist in Spain for the defense and security of the country are the Armed Forces (*Fuerzas Armadas*: FFAA), the State Security Forces and Corps (*Fuerzas y Cuerpos de Seguridad del Estado*: FFCCSE), and the Security Forces and Corps belonging to the Ministries of the Interior and Defense (*Fuerzas y Cuerpos de Seguridad pertenecientes a los Ministerios del Interior y de Defensa*: FFCCS).

The particular conditions and characteristics of these institutions of the Spanish State intervene in the process of management of the psycho-emotional state of professionals. An overloaded context with too much work and emotional burden can have negative repercussions on their state of mental health [1,2,3,4,5], both in the short and long term, as has been shown in recent studies [6,7,8,9]. In the context of a pandemic such as that caused by the SARS-CoV-2 coronavirus, the agents of the Armed Forces, FFCCSE and FFCCS, have played a fundamental role in the protection and safety of citizens, such as the setting up of field hospitals, the disinfection of centers, health care or the transfer of patients or bodies. Although this profession is characterized by undertaking in an agile and efficient way actions that require readiness and skill, the continuous contact with the disease, exposure to the virus and death, in excessive and permanent conditions, has generated an emotional context to trigger the need for psychological help.

Since the beginning of civilization as we know it, pandemics have been occurring incessantly [10]. Over the past centuries, we have seen how certain viruses such as smallpox, measles, plague, cholera, typhoid, the so-called “Spanish flu”, AIDS, Ebola, or Zika have ravaged society, especially in the most vulnerable areas. However, one of the most virulent has been the spread of SARS-CoV2, whose origin is still unknown and whose vaccine is still in the process of gestation.

Globalization has helped the viruses that once left many infected and dead in specific areas of the population to spread very easily in recent times. As of September 22nd, the world figures according to the WHO [11] are 31,174,627 confirmed cases and 962,613 deaths, of which 640,040 cases are people in Spain and 30,495 deaths according to official figures from the Ministry of Health (*Ministerio de Sanidad*) [12], although various estimates from other organizations, such as the Carlos III Institute (*Instituto Carlos III*), the National Institute of Statistics (*Instituto Nacional de Estadística*: INE) or the Spanish Association of Funeral Professionals and Services (*Asociación Española de Profesionales y Servicios Funerarios*) considerably increase these deaths [13].

Being aware of the health, economic, and psychosocial consequences of past pandemics has produced a certain degree of tension in Spanish society, especially after the establishment of the alarm decree in March 2020 for the management of the health crisis derived from COVID-19 [14]. This situation is especially aggravated in those so-called essential professions [15] that have been working in the front line, sometimes without the minimum health and hygiene conditions typical of those moments of the health crisis.

In this context, with these working circumstances, where the lack of Personal Protective Equipment (PPE), hydroalcoholic gels, gloves, and PCR tests was evident, especially at the beginning of the pandemic, the professions related to the emergency services and security, which are the subject of this article, were included in the groups with a low probability of exposure to SARS-CoV-2 virus by the Ministry of Health [16]. This situation, together with the new action protocols and the continuous exposure to the virus and death, has led to an increased risk of stress [17] and death anxiety [18] in these professionals of the FFAA, FFCCSE, and FFCCS.

The Spanish management of the health crisis has been recently questioned by Spanish researchers who requested in The Lancet [19] the need to implement an external audit, to evaluate the errors and avoid their recurrence in a second wave, which is currently being entered. Furthermore, another recent study identifies that the lack of follow-up on infections and the lack of clear criteria when making decisions could have had a significant influence on the evolution of the pandemic in Spain [20] and especially on the management of stress and anxiety among professionals who had to deal with the most negative effects of the COVID-19.

In the recent scientific literature related to COVID-19, many authors have expressed the need to provide psychological care to essential professionals due to the high risk for their mental health, which can lead to disorders such as depression, exhaustion, anxiety, or post-traumatic stress disorder [21,22,23]. One of the most evident has been anxiety in the face of death [18], which in the case of agents who watch over the safety of citizens, has been greatly compromised because they have been in continuous contact with the health professionals [7]. However, the procedures for the prevention of occupational risks about SARS-CoV-2 [16] do not take into account actions to alleviate the effects on mental health and the prevention of psychosocial risks that could have a negative influence on the development of their work.

Death continues to be one of the elements of life that cause most fear in people, so this means: disappearance, destruction, annihilation, or oblivion [24,25]. It is a fact present in all branches of human knowledge [26]. The emotions that arise from this situation serve as a support for the process of acceptance, but in the face of an overwhelming situation and without the appropriate psychological resources, it can become a focus of emotional distress and disturbance.

Death anxiety has been studied in recent decades as part of the prevention process of some professions, especially those called welfare and essential. Authors as Lewis [27] assert that the anxiety in the face of death supposes an annoying emotional state in which the sensation of death is experienced united to different physical manifestations, like the sensation of drowning, difficulty breathing, oppression in the chest, vomits, or tremors, among others.

These experiences can unleash a preventive attitude in the face of a possible threat that generates such tension that it can end up de-structuring the life of the professional [28], developing symptoms related, on the one hand, to those of anxiety, and on the other, to the aspects that cause them (that which is related to the representation of the death of others as well as one’s own).

In this sense, the manifestations of anxiety are diverse and are classified into several groups Sierra et al. [29]: physical, among which are the cardiac, digestive, muscular, psychological, behavioral, cognitive, and social. These manifestations of anxiety can interfere in the daily work performance of people since their consequences vary between the sensation of dizziness, difficulty to take decisions, distrust, difficulty to be in rest, susceptibility, alteration in the processes of attention, concentration, memory, confusion, or difficulty to initiate or to maintain a conversation.

Anxiety in the face of death does not manifest itself in a concrete way that can be identified instantly; it has difficulty in recognizing the exact cause that produces this uneasiness because the origin of fear can reside in multiple causes. It is believed that the behavior and reactions to the virus fall within the theory of terror, which argues that the fear of death is the driving force behind much of human behavior [30,31,32,33,34,35]. That is why the authors Collett and Lester [36] created the Fear of Death scale, which distinguishes four major components: fear of one’s death, fear of the death of others, fear of the process of one’s death, and fear of the process of death of others. This is the reason why this scale was used to develop the methodological process of the present study.

## 2. Materials and Methods

### 2.1. Objectives

The objectives of this research are two-fold. In the first place, (PO1) it is intended to learn the level of anxiety in the face of death of members of the State Security Forces and Corps and the Armed Forces. In the second place, (PO2) it is sought to determine what are the predictive variables of the phenomenon of suffering anxiety in the fac of death of these professionals. In both cases, an approach to anxiety is made both in its general index and in its respective subscales according to the Collett–Lester Death Anxiety Scale [36].

### 2.2. Variables

#### 2.2.1. Dependent Variable

In the approach to the object of study, the Collett–Lester Fear of Death Scale was used [36], validated by Venegas et al. This scale, adapted to Spanish, is formed by four subscales: “Fear of one’s death,” “Fear of one’s process of dying,” “Fear of the death of others,” and “Fear of the process of dying of others.” In this way, an approach to the phenomenon of death anxiety is made from a multidimensional perspective. Although there are other scales to measure different variables related to death such as the *Bugen’s Coping with Death Scale* [37] used in previous studies [38], the *Death Anxiety Clinical Scale* (DACS) [39] or the Death Anxiety Inventory (DAI) [40], The Collett–Lester Fear of Death Scale was chosen because it contains subscales that would allow a more in-depth understanding of the fear of death of other non-health professionals, such as police officers, where this scale was previously administered [41]. The response options are distributed on a Likert-type scale from 1 (nothing) to 5 (a lot). The values of the superior average subscales indicate high levels of anxiety in the face of death and those that inferior suppose low levels of anxiety. Concerning the total value of anxiety before death, it is constructed from the average value of the subjects in the subscales.

#### 2.2.2. Independent Variables

Three types of independent variables were established: (a) socio-demographic, (b) subjective perceptions of the current situation at work, and (c) the Maslach Burnout Inventory (MBI) subscales [35,36]. With respect to the sociodemographic variables, the following are used: sex (female/male), age (up to 30 years old, 31–40 years old, 41–50 years old, 51–60 years old, and over 60 years old), professional category (military of the Armed Forces, National Police and Guardia Civil) and whether they worked during the first wave of the pandemic (Yes/No). The following subjective variables were used: the need for psychological treatment (Yes/No), need to incorporate psychological/psychiatric treatment (Yes/No), assessment of the personal need for future psychological treatment (Yes/No), and assessment of how the lack of PPE may be affecting his/her level of stress or anxiety (Yes/No) and whether he/she has felt recognized by the organization to which he/she belongs (Yes/No). Finally, about the MBI, its three subscales were used: Emotional Exhaustion (EE), Depersonalization (DP), and Personal Accomplishment (PA). These variables were established dichotomously based on low or medium/high values. This inventory has been used to establish the incidence of burnout in the security forces in previous studies in different countries, thus justifying its validity [42,43].

### 2.3. Participants

Concerning the participants in the investigation, the number of participants reached 2079 (N = 2079), distributed among 374 military personnel of the Armed Forces, 800 National Polices (*Policía Nacional*), and 905 Civil Guards (*Guardia Civil*). The main characteristics of the participants in the research are shown in Table 1. The majority of the sample is made up of men, who represent 87.5% of the total. Women represent only 12.5%, a sign of a much-masculinized profile. In terms of age, the majority group is between 31 and 40 years old, who represent 35.2%, followed by people between 41 and 50 years old with 34.4%. In third place are people up to 30 years old. A lower level shows people between 51 and 60 years old and those over 60 years old with 18.5% and 0.9%. With respect to the professional category, 43.5% are Guardia Civil, 38.5% are National Police, and 18.8% are members of the Armed Forces. Of these, 73.2% worked directly in the first wave of the pandemic in COVID-19 contexts (Table 1).

### 2.4. Procedure

The field research has been carried out between 7 August and 7 September 2020. Spanish professional associations and trade unions agreed to collaborate in the research and administer the questionnaire. These entities distributed the questionnaire in a telematic way so that they could be completed when the participant considered it appropriate—not having the work environment as an intervening variable—through a survey application designed for this purpose that allows the completion and exploitation of results.

Although numerous similar empirical studies present a response rate, in this case, we cannot obtain such a record since this study does not emanate from the public administrations for whom these professionals work. On the other hand, there is also no record of the agglutination of all the participants (universe). For this reason, we obtained a sample from the entities and associations that voluntarily decided to collaborate in this research by facilitating the administration of the survey, understanding the importance of knowing how the first wave of the pandemic affected these professional bodies that worked directly with COVID-19.

On the other hand, a control group has not been established but a question was incorporated into the questionnaire that differentiates those who worked directly with COVID-19 from those who did not: “Worked with COVID-19 during the first wave of the pandemic.” In this way, we can see if there were differences in both according to their direct work with COVID-19, or not, in the first wave of the pandemic in Spain.

In this study, it has not been necessary for the official approval of the Spanish universities since it is a descriptive study (it is only required in the experimental works). However, the Codes of Good Practice for Research on Human Beings were signed, which are collected by the Ethics Committees and the study was registered (code No. REPRIN-PEM-15) by the research team that made up the authors. The participants (N = 2079) gave their informed consent under the Declaration of Helsinki.

The data exploitation and analysis process were carried out through the statistical program IBM SPPS V. 24 and two phases. Initially, a descriptive analysis was carried out to know the levels of anxiety before death, both of the different subscales and its general index. Later, to know the predictive variables in the phenomenon, binary logistic regressions were carried out, taking as dependent variables the existence of the high level of anxiety before death in its general index, as well as in its different subscales.

## 3. Results

### 3.1. Descriptive Results of the Research

**Figure 1:**
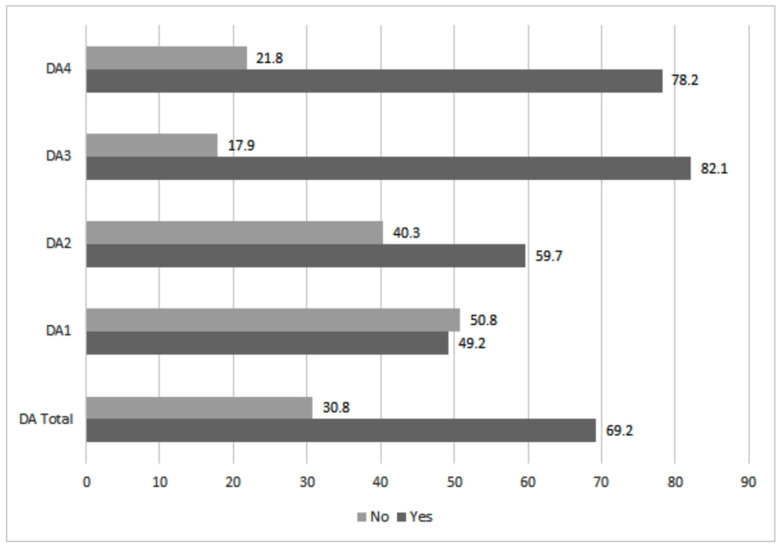
General index of DA and its subscales, in %.

Firstly, if an approximation to the DA (Death Anxiety) scale is made, in its general index as in the set of subscales the following results are observed. Concerning Fear of Death (DA1), 49.2% register positive values in this subscale, almost half of the participants. Higher percentages are obtained about Fear of one’s Process of Dying (DA2), reaching 59.7%. However, the highest values are obtained about the Fear of Death of others (DA3) and the Fear of the Process of Dying of others (DA 4). In the first case, 82.1% show fear with the death of others and 78.2% concerning the process of dying of others. Therefore, higher values are obtained for DA linked to the death and dying processes of others, much higher than if one asks about one’s death and dying process. As for Total DA, it reaches 69.2%, more than 2 out of every 3 professionals out of 2079 in the sample with this type of anxiety (Figure 1).

The following results have been obtained for the independent variables. First, according to the MBI subscales used, 53.8% show higher levels of Emotional Exhaustion. A higher percentage is obtained in the case of Depersonalization, whose higher levels rise to 58.4%, and in this case, which is more worrying, if the high and medium levels of the subscale are taken into consideration, they rise to 82.5%. As for Personal Accomplishment, it registers low levels -the reference levels in this subscale- of 27.0%, showing in this case a lower incidence. Concerning total MBI, 28.9% shows burnout, that is, they register high values in the first two subscales and low values in the last one.

With respect to the variables of subjective categories, 26.4% state that they currently need psychological or psychiatric treatment; however, 52.6% feel that they may need these services if we enter a new wave of the pandemic, a sign of the vulnerability of these people. On the other hand, 88.2% consider that workplaces should offer psychological or psychiatric treatment in workplaces as a consequence of the COVID-19. Besides, 87.8% stated that the absence of PPE increased levels of stress and anxiety during the first wave of the pandemic and 90.3% did not feel represented by the organization they work for (Table 2).

### 3.2. Predictive Variables in the Death Anxiety Phenomenon According to the Binary Logistic Regression Model

Subsequently, the technique of binary logistic regression was applied to each of the DA subscales as well as their total value.

#### 3.2.1. Fear of Death (DA1) Subscale

In the case of DA 1, the model was statistically significant x^2^ = 227.789, *p* < 0.000. The model explains 13.5% (Nagelkerke’s R^2^) of the variance of moderately high consumption and correctly classifies 63.6% of the cases. The Hosmer–Lemeshow test showed that there were no significant differences between the observed and predicted results in the model with a *p* = 0.535.

As for the variables predicting the DA 1 event, the following were significant: (a) PNPP, (b) PPE, and (c) Burnout Total, EE, and DP.

In the specific case of if PNPP presents an OR = 2.005, IC95% (1.662 to 2.418), *p* = 0.000. That is, a person who currently perceives that in the future, if the situation experienced during the first wave of the pandemic repeats itself, may need psychological or psychiatric treatment, is twice as likely to suffer from DA 1 like the rest. With respect to PID, he presents an OR = 1.629, IC95% (1.210 to 2.192), *p* = 0.001. Thus, people who consider that the lack of PID was a reason for their increased stress and anxiety, are 1.6 times more likely to suffer from DA 1. This situation is connected with burnout; those who suffer burnout have 1.4 times more suffering of DA 1. Within the burnout, there are two representative subscales. The first one, the EE presents an OR = 1.873, IC95% (1.494 to 2.347), *p* = 0.000. Therefore, the EE is connected to DA 1 so that those who have representative values in this subscale of the MBI have almost 2 times more suffering of DA 1. Finally, within DA 1, people who suffer from PD are almost 1.5 times more likely to suffer from this type of anxiety than those who have low values.

#### 3.2.2. Fear of One’s Process of Dying (DA2) Subscale 

In the case of DA 2, the model was statistically significant x^2^ = 196.301, *p* < 0.000. The model explains 11.9% (Nagelkerke’s R2) of the variance of moderately high consumption and correctly classifies 64.6% of the cases. The Hosmer–Lemeshow test showed that there were no significant differences between the observed and predicted results in the model with a *p* = 0.840.

As for the variables predicting the DA2 event, the following were significant: (a) PNPP, (b) PPE, and (c) Burnout Total and EE. All of these were also predictor variables in DA 1.

In the specific case of subjective perception of PNPP, it presents an OR = 1.864, IC95% (1.542 to 2.254), *p* = 0.000; thus, it increases by almost two times the possibilities of suffering DA2. The PPE variable presents an OR = 1.399, IC95% (1.058 to 1.849), *p* = 0.019. In this case, it also increases the possibility of suffering this phenomenon by 1.4 times. This same predictive level is recorded in the variable burnout Total, which presents an OR = 1.464, IC95% (1.167 to 1.836), *p* = 0.001. Finally, EE is the variable with the greatest predictive power, with an OR = 2.099, IC95% (1.692 to 2.605), *p* = 0.000; that is, people who suffer EE have up to 2.1 times more suffering from DA 2 than the rest.

#### 3.2.3. Fear of Death of Others (DA3) Subscale

In the case of DA3, the statistically significant model x^2^ = 150.491, *p* < 0.000. The model explains 11.2% (Nagelkerke’s R^2^) of the variance of moderate-high consumption and correctly classifies 82.4% of the cases. The Hosmer–Lemeshow test showed that there were no significant differences between the observed and predicted results in the model with a *p* = 0.965.

As for the variables predicting the DA3 event, the following variables were significant: (a) PNPP, (b) PPE, (c) EE, (d) DP, and (e) age (41–50 and 51–60).

In the specific case of subjective perception of PNPP it presents an OR = 1.559, IC95% (1.206 to 2.017), *p* = 0.001; thus, this variable increases by 1.5 times the possibilities of suffering DA3. As for PID, it presents an OR = 1.888, IC95% (1.386 to 2.571), *p* = 0,000. In this case, it increases the possibility of suffering this phenomenon by almost 2 times. A similar predictive level is recorded in the variable EE, which presents an OR = 2.057, IC95% (1.601 to 2.643), *p* = 0.000. Regarding DP, it registers an OR = 1.494, IC95% (1.126 to 1.986), *p* = 0.005; that is, people who suffer from PD have up to 1.5 times more possibilities of suffering DA3 than the rest.

Special mention for the socio-demographic variable age is its subcategories of 41–50 and 51–60 years. The cohort between 41 and 50 years presents an OR = 1.442, IC95% (1.047 to 1.987), *p* = 0.025. On the other hand, those whose age ranges between 51 and 60 years register an OR = 2.084, IC95% (1.320 to 3.288), *p* = 0.002. That is, those between 41 and 50 and 51 and 60 years old have almost 1.5 and 2.1 times more DA 3 than their reference value (up to 30 years old).

#### 3.2.4. Fear of the Process of Dying of Others (DA4) Subscale

In the case of DA4, the statistically significant model x^2^ = 150.491, *p* < 0.000. The model explains 10.9% (Nagelkerke’s R^2^) of the variance of moderate-high consumption and correctly classifies 78.3% of the cases. The Hosmer–Lemeshow test showed that there were no significant differences between the observed and predicted results in the model with a *p* = 0.445.

As for the variables predicting the DA4 event, the following variables were significant: (a) PNPP, (b) PPE, (c) EE, (d) DP, and (e) the fact of not having worked during the first wave of the pandemic.

In the specific case of subjective perception of PNPP, it presents an OR = 1.758, IC95% (1.398 to 2.211), *p* = 0.000; thus, this variable increases by 1.7 times the possibilities of suffering DA4. As for PID it presents an OR = 1.517, IC95% (1.134 to 2.056), *p* = 0.005. In this case, it increases the chances of suffering this phenomenon by 1.5 times. The highest predictive level is recorded in the variable EE, which presents an OR = 2.444, IC95% (1.937 to 3.084), *p* = 0.000; therefore, these people would be almost 2.5 times more likely to suffer DA4 than the rest. Regarding DP, it registers an OR = 1.359, IC95% (1.037 to 1.780), *p* = 0.026; that is, people who suffer from DP have up to 1.3 times more possibilities of suffering from DA4. Relative to people who worked directly with the pandemic, they show an OR = 0.716, 95% CI (0.556 to 0.922), *p* = 0.010; however, their B is negative (−0.335). Thus, even slightly, people who worked directly with the COVID-19 are less at risk for DA4. An explanation can be found in the psychological adaptation to the risk of contagion and contact with mortality, in these professionals, there is a compensatory effect of the risk of suffering DA4.

#### 3.2.5. Death Anxiety Total

In the case of Total DA, the statistically significant model x^2^ = 221.588, *p* < 0.000. The model explains 13.9% (Nagelkerke’s R^2^) of the variance of moderately high consumption and correctly classifies 74.1% of the cases. The Hosmer–Lemeshow test showed that there were no significant differences between the observed and predicted results in the model with a *p* = 0.807.

The variables predicting the event DA Total are: (a) PNPP, (b) PPE, (c) EE, (d) DP, and (e) burnout total. In the specific case of subjective perception of PNPP, it presents an OR = 1.820, IC95% (1.482 to 2.234), *p* = 0.000; in this way, this variable increases by 1.8 times the possibilities of suffering DA Total. As for PPE it presents an OR = 1.617, IC95% (1.220 to 2.144), *p* = 0.001. In this case, it increases the possibility of suffering this phenomenon by 1.6 times. The highest predictive level is again recorded in the variable EE, which presents an OR = 2.221, IC95% (1.770 to 2.786), *p* = 0.000; therefore, these people would be almost 2.2 times more likely to suffer from Total DA. Regarding DP, it registers an OR = 1.383, IC95% (1.074 to 1.781), *p* = 0.012; that is, people who suffer from PD have up to 1.3 times more possibilities of suffering from DA Total. Finally, regarding burnout total, it records an OR = 1.444, IC95% (1.112 to 1.875), *p* = 0.006; therefore, the prediction of this variable in DA Total increases by almost 1.5 times (Table 3).

## 4. Discussion

The entry into the second wave of the pandemic in Spain, caused by the SARS-CoV-2 coronavirus, is bringing essential workers back into contact with the traumatic events that took place only a few months ago. While it is true that there is already more knowledge about the hygiene and health measures that could serve to avoid contact with the virus, the new outbreaks represent a higher level of stress due to the fear of experiencing new confinement with the consequences that the first one has brought, as well as seeing an increase in the number of daily deaths as occurred in the preceding months between March and May 2020.

This study shows that 52.6% of the members of the FFAA, FFCCSSE, and FFCCS feel that they may need psychological help if they enter a new wave of the pandemic, more than those who said they needed it during the most critical months of the pandemic, 26.4%, a sign of the vulnerability that these workers manifest; 88.2% believe that workplaces should offer psychological or psychiatric treatment. Studies in other countries have reached the same conclusion regarding the need for psychological intervention [35,44,45,46,47,48,49].

It should be noted that although death anxiety has a significant component related to the loss of one’s life, the results of the professionals of the FFAA, FFCCSSE, FFCCS have indicated that the highest values obtained have been with the fear of death of others and the process of dying of others so that the essential social concern during the pandemic has been higher than the personnel, which also occurs with other groups such as health [18]. Especially between the ages of 41–50 years and 51–60 years who had almost 1.5 and 2.1 times more DA3 than their reference value (up to 30 years). Therefore, as age increases, the risk of suffering DA3 during the context of the first wave of the pandemic increases, which could be in line with data from the *Instituto de Salud Carlos III* [50] that showed the risk of presenting dangerous symptoms caused by COVID-19 and the higher number of deaths increased with age.

Despite this, levels of depersonalization amount to 82.5%, very high levels that show that stress may be affecting the proper performance of their daily work, especially at critical times such as a pandemic, as noted by other studies [51,52,53,54,55], and which also extends to other professions who have played a crucial role as well as healthcare providers [56,57] showing that these so-called “essential” professions have suffered a great impact on their professional and personal well-being due to the existence of COVID-19.

The present study has shown that 87.8% say that the absence of PPE increased the level of stress and anxiety during the first wave of the pandemic. Not surprisingly, the number of professionals affected in one way or another rose rapidly because of this lack of protection, as was also the case in other countries such as Peru [58].

On the other hand, the set of binary logistic regressions carried out shows that there are clear predictive variables in the phenomena of Total DA, as well as in the set of its subscales. These variables are PNPP, PPE, EE, and DP. In other words, death anxiety is mainly conditioned by the subjective perception of needing psychological or psychiatric treatment, an element to be taken into account, since it retraces the perceptions, feelings, and dwellings that occurred in these professionals during the first wave of the pandemic. Secondly, the lack of PPE, since one of the characteristics these professionals had to face was the absence of essential means to protect themselves, the people they worked with, and, by extension, their families. Thirdly, anxiety in the face of death is connected to the burnout they suffer. The subscales of EE and DP can be considered the most representative. That is, despite the complicated situations in which they worked, their work commitment has not been diminished and PA does not act as a predictive variable.

One fact that is worth noting is that although the results have shown that the professionals of the FFAA, FCCSSE, and FFCCS have focused not only on their professional work of which they show high personal achievement, despite the high percentages of mortality among their colleagues and the civilian population, but also on the fear of death of those they protect, and despite that, 90.3% have not felt represented by the organization they work for. One explanation could be that in recent times there have been different positions between the government of the nation and the entities in charge of ensuring the security and protection of the country in different matters such as wage equality, the non-recognition of them as high-risk personnel during the pandemic, the controversial dismissal of a colonel as the head of the Madrid Command of the Guardia Civil [59], or the dismissal of the chief of police who drafted the protocol against the coronavirus [60]. These aspects produce a distancing and even a rupture with those in charge of the Corps, which runs a double risk: on the one hand, the emotional distancing of the people with whom they work and the repercussions of possible malpractice (in keeping with the results obtained concerning depersonalization, which favors increased dehumanization when working with others), and on the other hand, a lack of identification with the organization, both with its internal structure from a hierarchical level and with the functions it performs for the benefit of citizens. In this sense, previous studies in the field of Social and Organizational Psychology show that involvement with work can increase the consequences of all occupational stressors and, nevertheless, its modulating influence can be conditioned by the stressors that impede the exercise of the successful role [61], that is, two of the dimensions proposed by Lodhal and Kejner [62] about occupational involvement as the psychological identification with the work being performed decreases the burnout, but the duty-obligation increases it [63].

However, even slightly, people who worked directly with COVID-19 are less likely to have DA4. One explanation can be found in the psychological and emotional adaptation to the risk of contagion and contact with mortality in these professionals, which produces a compensatory effect of the risk of suffering DA4.

But it should not be forgotten that the pandemic has produced important daily changes in the social and family life of essential workers such as the FFAA, FFCCSE, and FFCCS, which are closely related to the possibility of illness and death, such as concerns about one’s health, fear of bringing the infection home and infecting family members, which sometimes meant isolation, changes in work routines and adaptation to them, which could explain certain states of stress, loneliness, and uncertainty that would affect the proper development of their professional practice [7,18,35,64].

The short and medium-term effects can be very serious [64,65,66,67], given that both the context in which they carry out their professional practice and the development of their functions occur in an environment that facilitates the development of high anxiety in the face of death [18,35,68,69,70]. These factors may condition the response of these professionals, running the risk of producing an institutional separation of these professionals, precisely those who have the obligation and duty to look after the collective interests of the citizens in moments of crisis and catastrophes such as the present one.

In the long term, this health crisis could help to understand and anticipate the risk factors associated with the mental health of essential workers who have been on the front lines during the months of the pandemic. This can help develop preventive public health strategies both in the health crisis arising from COVID-19 and in subsequent outbreaks where an effective and prompt response is required [64].

The abnormal circumstances that not only Spain but the rest of the world is experiencing, due to the health crisis and the subsequent social and personal crises caused by the SARS-CoV-2 coronavirus, is making clear the need for improvement in all spheres of political and social life. The dedication of the institutions for the protection of citizens and the professionals who work for their security (FFAA, FFCCSSE, FFCCS, and health personnel), has been greatly compromised in light of the events and the requests of the associations to which these groups belong.

In this sense, the increase in burnout and anxiety in the face of the death of defense and security professionals due to the conditions in which they have been forced to carry out their work, especially in the first months of the pandemic, has caused a demand for psychological resources to be able to deal with post-traumatic disorders and to prepare for other circumstances that may be just as stressful as the previous ones. For this reason, these groups must develop programs to reduce anxiety in the face of death and stress and to improve emotional intelligence, so that they can be applied correctly in circumstances such as those that humanity, as a whole, is currently experiencing.

This research has encountered some limitations. One of them has been the difficulty in obtaining the sample due to the continuous and self-sacrificing work of the members of the Armed Forces, FFCCSSE, and FFCCS, who hardly have time to participate in research of this nature. This could be the reason why there are few studies of this group related to the subject matter of COVID-19. In our case, the professional associations have contributed decisively to making it a reality. Another limitation has been the difficulty of comparing studies with the same group in other countries due to the lack of contextualized studies at this stage of the pandemic.

## Figures and Tables

**Table 1:** Sociodemographic characteristics of the participants.

Title	%
**Gender**	
Woman	12.5
Man	87.5
**Age**	
Up to 30	18.5
31–40	35.2
41–50	34.4
51–60	11.1
>60	0.9
**Work**	
FFAA	18.0
Guardia Civil	43.5
Policía Nacional	38.5
**Worked with COVID-19 during the First Wave of the Pandemic**	
Yes	73.2
No	26.8

**Table 2:** Descriptive results of the independent variables.

**Sub Emotional Exhaustion (EE)**	
Low	29.8
Medium	16.4
High	53.8
**Sub Depersonalization (DP)**	
Low	17.5
Medium	24.1
High	58.4
**Sub Personal Accomplishment (PA)**	
Low	46.8
Medium	26.2
High	27.0
**Total MBI**	
Yes	28.9
No	71.1
**Need Psychological or Psychiatric Support (NAPP)**	
Yes	26.4
No	73.6
**Psychological or Psychiatric Support from the Workplace (APPC)**	
Yes	88.2
No	11.8
**May Need Psychological or Psychiatric Support (PNPP)**	
Yes	52.6
No	47.4
**Lack of PPE Increased Your Stress and Anxiety Level (PPE)**	
Yes	87.8
No	12.2
**Feel that Your Work Has Been Recognized by Your Institution (SRI)**	
Yes	90.3
No	9.7

**Table 3:** Summary of binary logistic regression models.

	B	Sig.	Exp (B)	95% C.I. Exp (B)	
				Lower	Superior
**DA 1**					
PNPP	0.696	0.000	2.005	1.662	2.418
PPE	0.488	0.001	1.629	1.210	2.192
Total Burnout	0.347	0.002	1.414	1.137	1.760
EE	0.627	0.000	1.873	1.494	2.347
DP	0.391	0.003	1.478	1.141	1.914
Constant	−1.706	0.000	0.182		
**DA 2**					
PNPP	0.623	0.000	1.864	1.542	2.254
PPE	0.335	0.019	1.399	1.058	1.849
Total Burnout	0.381	0.001	1.464	1.167	1.836
EE	0.742	0.000	2.099	1.692	2.605
Constant	−0.847	0.000	0.429		
**DA 3**					
41-50 years old	0.366	0.025	1.442	1.047	1.987
51-60 years old	0.734	0.002	2.084	1.320	3.288
PNPP	0.444	0.001	1.559	1.206	2.017
PPE	0.635	0.000	1.888	1.386	2.571
EE	0.721	0.000	2.057	1.601	2.643
DP	0.402	0.005	1.494	1.126	1.983
Constant	−0.564	0.016	0.569		
**DA 4**					
PNPP	0.564	0.000	1.758	1.398	2.211
PPE	0.423	0.005	1.527	1.134	2.056
Worked COVID-19	−0.335	0.010	0.716	0.556	0.922
EE	0.894	0.000	2.444	1.937	3.084
DP	0.307	0.026	1.359	1.037	1.780
Constant	0.073	0.685	1.076		
**DA Total**					
PNPP	0.599	0.000	1.820	1.482	2.234
PPE	0.481	0.001	1.617	1.220	2.144
Total Burnout	0.367	0.006	1.444	1.112	1.875
EE	0.798	0.000	2.221	1.770	2.786
DP	0.324	0.012	1.383	1.074	1.781
Constant	−0.781	0.000	0.458		
